# Design and Analysis of a Low Latency Deterministic Network MAC for Wireless Sensor Networks

**DOI:** 10.3390/s17102185

**Published:** 2017-09-22

**Authors:** Prasan Kumar Sahoo, Sudhir Ranjan Pattanaik, Shih-Lin Wu

**Affiliations:** 1 Department of Computer Science and Information Engineering, Chang Gung University, Taoyuan 33302, Taiwan; pksahoo@mail.cgu.edu.tw; 2 Department of Cardiology, Chang Gung Memorial Hospital, Taoyuan 33305, Taiwan; 3 Department of Electrical Engineering, Chang Gung University, Taoyuan 33302, Taiwan; sudhirpattanaik.oca@gmail.com; 4 Department of Electrical Engineering, Ming Chi University of Technology, New Taipei City 24301, Taiwan

**Keywords:** wireless sensor networks, IEEE 802.15.4e, performance analysis, LLDN, star topology

## Abstract

The IEEE 802.15.4e standard has four different superframe structures for different applications. Use of a low latency deterministic network (LLDN) superframe for the wireless sensor network is one of them, which can operate in a star topology. In this paper, a new channel access mechanism for IEEE 802.15.4e-based LLDN shared slots is proposed, and analytical models are designed based on this channel access mechanism. A prediction model is designed to estimate the possible number of retransmission slots based on the number of failed transmissions. Performance analysis in terms of data transmission reliability, delay, throughput and energy consumption are provided based on our proposed designs. Our designs are validated for simulation and analytical results, and it is observed that the simulation results well match with the analytical ones. Besides, our designs are compared with the IEEE 802.15.4 MAC mechanism, and it is shown that ours outperforms in terms of throughput, energy consumption, delay and reliability.

## 1. Introduction

The IEEE 802.15.4 [1] standard is popular for short-range and low-power wireless networks, which supports sixteen channels in 2.4-GHz and ten channels in 902-/928-MHz Industrial, Scientific and Medical (ISM) band. It specifies the physical and medium access control (MAC) layer and defines the format of the data handling. ZigBee is an enhancement to the IEEE 802.15.4 that supports the network to application layer. Integration of ZigBee with IEEE 802.15.4 is popular because of its low power consumption, low bandwidth, low cost and ease to implement. It is expected that these ZigBee sensors will play a major role in different applications of IoT. However, the critical requirements in the industrial/commercial IoT applications such as high reliability and low energy consumption in the industrial environment have not been addressed adequately in the IEEE 802.15.4 standard.

The IEEE 802.15.4 standard defines the slotted and unslotted channel access mechanism through carrier sense multiple access with collision avoidance (CSMA/CA) for communication. To start a transmission, each IEEE 802.15.4-enabled node first performs clear channel assessment (CCA) to ensure that the channel is idle. The IEEE 802.15.4e [2] working group has redesigned the existing IEEE 802.15.4 MAC protocol to overcome the limitations such as low latency and robustness to meet the critical requirements of IoT applications. They define a low-power multi-hop MAC protocol, which is capable of addressing the emerging needs of different IoT applications. The final standard of the IEEE 802.15.4e MAC enhancement protocol adopts the ideas like slotted access, multi-channel communications and frequency hopping from WirelessHART [3] and International Society of Automation (ISA) 100.11.a [4].

IEEE 802.15.4e supports low latency deterministic network (LLDN) MAC, which can be applied to different applications like fall detection, factory automation, robots, automated dispensations, airport logistic and many emergent automated applications. The major requirements of such applications are to reduce the delay and energy consumption. It is to be noted that the sensing data of applications are typically of a few bytes, which make the time slot size very small. As a result, the sensing data from nodes can be accommodated in one superframe due to the small slot size. Allocating a dedicated time slot for each LLDN node in the superframe provides a deterministic system. Due to the strict requirements of such low-latency applications, LLDN uses a star topology with a minimal number of superframe structures. The star topology has a special node called the coordinator that manages communications among the nodes in a personal area network (PAN).

Various applications of wireless sensor networks (WSN) require low latency data transmissions. Therefore, if any sensor node fails to transmit its packet, a retransmission for the failed packet should be arranged within the current superframe to meet the low latency requirement. All retransmissions within an LLDN superframe are possible whenever the numbers of failed packet are less than or equal to the available retransmission slots. However, there is a problem that the numbers of failed packets are greater than the available retransmission slots. The PAN coordinator fixes the number of retransmission slots for a superframe and cannot be changed dynamically. Note that there is no provision of retransmissions in IEEE 802.15.4e LLDN in the case of the non-availability of retransmission slots, and as a result, the performance of the network in terms of delay, throughput and packet drop rate is degraded. Two types of slot allocations (dedicated or shared) are made for nodes attached to the coordinator in the LLDN superframe. In the case of shared slots, when a slot is not used by the slot owner, it can be used by other nodes associated with the slot through the CSMA/CA mechanism. As per the existing CSMA/CA mechanism of the IEEE 802.15.4e standard, a node has to go for the CCA twice in order to avoid the collision due to acknowledgment transmissions.

Normally, collisions occur in WSN either due to simultaneous channel assessment or a hidden terminal problem. When two nodes are not present in the communication range of each other and transmit to another node, which is present within both’s communication range, it is known as the hidden terminal problem. The hidden terminal problem can be avoided by allocating a common shared slot in the LLDN superframe to the nodes present within the same communication range and different shared slots to the hidden nodes. The random backoff and CCA in the CSMA/CA mechanism avoid the collisions and are only effective for the nodes that are not hidden. However, they cannot minimize the number of collisions due to simultaneous transmissions, and therefore, these problems need to be addressed for better channel utilization. To mitigate the problems, a new channel access mechanism is proposed in this paper.

The originalities in our work are that a new prediction model is designed to predict the optimal numbers of retransmission slots based on the historical data of the previous transmissions and performance analysis of different network parameters of the LLDN superframe such as reliability, throughput, energy consumption and delay, which have not been studied yet. The main contributions of our work can be summarized as follows.

- In order to reduce the power consumption of sensors, a new CSMA/CA mechanism is designed for the shared slots of the LLDN superframes.
- In order to avoid collision due to simultaneous transmission by nodes, a new channel access mechanism is designed.
- Mathematical models are designed to predict the optimal numbers of retransmission slots based on the historical data of the previous transmissions.

The rest of the paper is organized as follows. The survey of related works of the existing IEEE 802.15.4 standard are given in Section 2. The overview of LLDN superframe is given in Section 3. The network model and proposed MAC mechanisms are presented in Section 4. Various analytical models are designed in Section 5. The performances of various network parameters are studied in Section 6. Simulation results are given in Section 7, and concluding remarks are made in Section 8.

## 2. Related Works

A general approach to evaluate the IEEE 802.15.4 performance of slotted the CSMA/CA protocol for only the unsaturated traffic condition is designed in [5]. By considering both saturated and unsaturated traffic to predict the energy consumption, as well as throughput, the authors in [6] have presented an analytical model for the IEEE 802.15.4 MAC protocol. A Markov chain-based analytical model is introduced in [7] to evaluate the impact of throughput and energy consumption on the probability of delivering a packet. However, these protocols did not talk about how to allocate the retransmission slots in case of packet failures. The authors in [8] have designed one analytical model for the guaranteed time slot (GTS) allocation mechanism during the contention-free period (CFP). The authors in [9] have proposed a methodology to analyze the GTS mechanism in the CFP. The performance of IEEE 802.15.4 MAC is analyzed in [10] for both CFP and the contention access period (CAP). However, these works did not address transmission failures due to interference or collision, which is an important issue for the low latency applications. The authors in [11] have designed one additional carrier sensing algorithm to get information from the busy channel due to data or acknowledgment (ACK) transmissions during the second CCA. However, the power consumption will be more if the second CCA is found busy due to data/ACK transmissions. A new MAC protocol is designed in [12] to avoid the channel being busy due to acknowledgment packet transmission without any additional CCA. To increase the probability of data transmission by ignoring the first CCA channel busy condition due to ACK transmissions, the authors in [13] propose one segmentized CCA mechanism. However, there is no improvement in the case that a node transmits a data packet instead of an ACK.

The effects of different *macSuperframeOrder* (SO) values on the total network throughput, delay and energy consumption through simulation have been studied in [14]. However, the work entirely focuses on the ideal channel condition, which is not realistic because the channel condition oscillates between good and poor in the wireless environment. The contention-based protocols are widely used in WSN applications to reduce delay and collisions. The authors [15] have studied the stability and accuracy of the wireless technology by using the IEEE 802.15.4/ZigBee technology in monitoring human body temperature. However, the authors have not considered any retransmission opportunity for transmission failure. A mathematical model is designed in [16] based on stochastic geometry, which is used for performance evaluations of success probability in transmitting packets from nodes to the coordinator. However, the authors have not done the performance analysis such as delay and energy, which are crucial factors for some WSN applications. The impact of fading statistics on the MAC performance in terms of reliability, delay and power consumption by varying traffic rates, inter-nodes distances, carrier sensing range and the signal-to-interference-plus-noise ratio (SINR) threshold have been studied in [17]. However, all of these works are based on the IEEE 802.15.4 standard. The major requirements of industrial application are very low latency communication. Hence, the LLDN MAC mechanism of the IEEE 802.15.4e [2] standard is most suitable for these requirements.

Comparisons of different technical parameters between Bluetooth, ZigBee and Wi-Fi are given in [18], where the received signal strength indicator (RSSI) value, round trip delay (RTD) time and latency are analyzed. The performance of the network considering both high and low data rate is analyzed in [19]. The impact of retry limit, backoff, network lifetime under all different data rates and traffic loads is also analyzed in the same work. However, they have not taken the priority into account for the transmission failure by the nodes. A comprehensive analysis of energy consumption of body area networks including the effect of packet inter-arrival time are given in [20]. However, the work did not consider retransmissions within the current superframe. To enhance the reliability of the LLDN networks, the authors in [21] propose one retransmission scheme for the time-varying channels by choosing the best relay node through the reinforcement-learning method. A new MAC protocol is proposed in [22] to minimize the energy consumption in WSN, where the authors have not considered the transmission failure due to the channel error. To avoid external interference, different frequency adoption schemes are proposed in [23]. A priority-based adaptive time slot allocation method is proposed in [24] based on the IEEE 802.15.4 MAC protocol. However, they have not considered the transmission failure nodes.

The stability of the WSNs in terms of throughput is analyzed in [25] considering the exponential backoff scheme. However, the work is based on the IEEE 802.15.4 MAC mechanism. Collisions normally occur when the number of nodes increases in the network. To avoid the energy consumption due to collisions and to enhance the throughput of the network, the authors in [26] have proposed one collision-free MAC mechanism. However, they have not considered the retransmission opportunity for the transmission failure nodes. The hidden node problem increases the number of retransmissions in the network such that the battery lifetime of nodes is affected. Imperfect channel sensing is also one of the causes to significantly affect the network performance. To avoid the hidden node collisions, a new protocol is proposed in [27] by using carrier sense multiple access with collision notification (CSMA/CN) to approximate carrier sense multiple access with collision detection (CSMA/CD) for the wireless network. In this work, the authors use two antennas to emulate full duplex mode so that collision notifications can be allowed to broadcast, and the ongoing transmissions can be aborted. The use of transmission power control can help to address industrial issues concerning energy consumption, interference and fading. In [28], an adaptive multi-channel transmission power control algorithm for industrial wireless networks such as WirelessHART, ISA 100.11a has been designed RSSI. However, when a transmitted packet is not acknowledged properly, it needs to be retransmitted at the cost of additional energy and delay. Besides, the authors did not consider the transmission failure due to channel error and the retransmission opportunity in the current superframe to avoid the delay.

A Markov model is designed in [29] to analyze the throughput of the IEEE 802.15.4 network with the presence of hidden nodes. Almost all existing analytical models assume ideal channel conditions. However, in real-world scenarios, wireless channels exhibit burst errors. A three-dimensional Markov chain model is proposed in [30], which is applied to analyze the network performance under burst channel errors. Comparative studies between IEEE 802.15.4e LLDN and IEEE 802.15.4 slotted CSMA/CA have been analyzed in [31] under the ideal channel condition, which is not realistic. In [32], the authors evaluate the impact of losing synchronization under the beacon-enabled star topology of the IEEE 802.15.4 network. To avoid collisions in a dense network, a new variable CCA MAC protocol for WSNs is designed and the performance analyzed in [33]. However, this protocol needs a significant amount of energy, which is the major constraint for sensors.

From the current literature survey, we noticed that most works are based on the performance analysis of the IEEE 802.15.4 standard. To the best of our knowledge, the performance of LLDN has not been analyzed yet, which has many applications in home, healthcare and industry automation. In this paper, we not only propose a new channel access mechanism to avoid the collision due to simultaneous transmissions, but also design one CSMA/CA mechanism for the shared slots of LLDN superframes. In addition, one prediction model is proposed to predict the optimal number of required retransmission slots based on the historical data of the previous transmissions, and one analytical model is proposed to study the performance of different network parameters such as reliability, throughput, energy consumption and delay.

## 3. Overview of the LLDN Superframe

**Figure 1:**
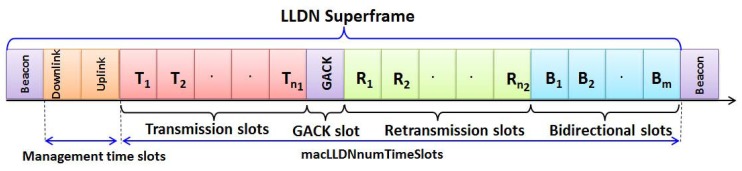
LLDN superframe structure with separate group acknowledgment (GACK).

Each superframe in IEEE 802.15.4e LLDN contains a beacon slot, management time slots and *macLLDNnumTimeSlots* of equal duration as shown in Figure 1. In the beginning beacon slot of each superframe, the PAN coordinator broadcasts the beacon frame, and the associated nodes start synchronization with the superframe structure. The beacon frame also helps the nodes for re-synchronization, which have gone due to entering the power saving mode. The time slots in the superframe can be assigned to one or more nodes. However, each slot is assigned to exactly one node and called the slot owner. The slot owner can transmit the data without any explicit addressing in the data frame. When more than one node is associated with one shared slot, it is known as the shared group time slot.

The nodes in a group shared slot follow the CSMA/CA mechanism with a simple eight-bit address inside the data frame to transmit the data to the coordinator. Management time slots are used to transmit management data in both the uplink and downlink. Apart from this, the presence of the bidirectional time slot gives scope for communication between the coordinator and nodes. However, the direction of communication is broadcast through the beacon. As shown in Figure 1, one slot is reserved to broadcast a separate group acknowledgment (GACK) by the coordinator, and only the failed nodes use the retransmission slots in the superframe. Note that a node does not know about the number of failure nodes. However, with the information received from the GACK that contains one bitmap to indicate the successful and failed transmissions, a node can choose the suitable retransmission slot in the same order as the transmission slot. Based on this information, only the collided nodes attached to one shared slot can find the suitable retransmission slots and access the channel through CSMA/CA.

## 4. Proposed MAC for LLDN

In this section, for collisions due to simultaneous transmissions, a new MAC protocol is designed. Below, we first give the network model, as well as our assumptions and then present our detailed MAC protocol.

### 4.1. Network Model

Consider a WSN of star topology, where *N* number of nodes, $W_{0},W_{1},\ldots .,W_{N}$, are associated with the PAN coordinator. Each node uses the CSMA/CA channel access mechanism in the allotted shared slot for competing to transmit data frames to the coordinator. The coordinator follows the LLDN superframe structure as shown in Figure 1. The owner nodes associated with dedicated slots have a better chance for successful transmissions. Assume that nodes always transmit the most recent generated data to the coordinator, and the sensing range of a node is twice of the transmission range. Every node goes for the power saving mode after the scheduled transmission/receiving slots. A node defers the transmission during accessing the channel in a shared slot if the remaining time is not enough to transmit the data in the shared slot.

### 4.2. Proposed CSMA/CA Mechanism for LLDN

Consider a node that tries to transmit a generated packet at a certain time. According to the procedure of the IEEE 802.15.4e MAC protocol, the node needs to wait for a random number of back-off periods and then goes for CCAs twice. The node is allowed to transmit data, if the channel is idle during both CCAs. Unfortunately, this procedure can cause collision due to simultaneous transmissions, if any two nodes generate the same random back-off period. Since a node remains unaware of the collision until the acknowledgment is received, it goes for the data transmission, which increases the energy consumption of the node. Our proposed approach wants to eliminate the simultaneous transmissions to avoid the collision, which can reduce energy consumption. According to the standard, one backoff slot is 20 symbol periods, and the CCA detection time requires eight symbol periods. One symbol is considered as 16μs, and the data rate is 250 kbps. Therefore, using the rest 12 symbols of a mini slot, we can send a signal of $\frac{12\;\times \;16\;\mu s}{32\;\mu s}≐6$ bytes. We use these remaining 12 symbols and design a uniquely computed known signal (KS) set of the six-byte uniquely-computed signal ($KS=\{KS^{1},KS^{2},KS^{3},\ldots ,KS^{m}\}$, and each element has the length of six bytes) for our proposed method to avoid the simultaneous collision. These known signals are the random patterns designed by the coordinator that distract from the information. In our proposed method, these six-byte signals acts like a preamble to sense the medium in order to know whether any other node is performing CCA at the same slot within the communication range of the node.

It is proposed that a node utilizes two antennas in which one is used for normal transmissions and the other one is dedicated to listening to the intended signal. The presence of a dual antenna in the sensor may increase the hardware cost. However, it reduces the power consumption significantly [27]. The concept of using two antennas in our protocol is similar to the idea given in [27], but the power consumption of our protocol is less than [27], as both antennas in our design are active during the CCA period only instead of being fully active throughout the communication duration in [27].

When a node has a packet to transmit, it goes for the random backoff and CCA. During the first 128 μs of the mini slot, a node can find the channel busy if any other node is transmitting at the same time. The node can enable its transmitter after 128 μs of the CCA, and sends one random six-byte signal from KS (say $KS^{k}$). All nodes performing CCA at the same time also transmit one random signal. The node of the received signal in the same slot will calculate the signal correlation with the rest of the signals in KS. This signal correlation is the optimal technique [27] for detecting a known waveform in the environment with random noise.

Let X[n] be the complex number representing the *n*-th transmitted symbol and Y[n] be the complex number representing the *n*-th received symbol. Then:

(1) Y[n]=H×X[n]+ω[n]

where *H* is a complex number representing the channel coefficient between the transmitter and the receiver and ω[n] is the random noise. The cross-correlation known symbol pattern of length *L* in the received signal *Y* at a shifted length *l* is:

(2) $$C\left(KS^{j},Y,l\right)=\sum_{i=1}^{l}\bar{KS^{j}\left[i\right]}\times Y\left[i+l\right],\;for\;all\;j\in \left[1,m\right]\;and\;j\neq k.$$

where $\bar{KS^{j}\left[i\right]}$ is the complex conjugate of $KS^{j}\left[i\right]$. The cross-correlation value $C(KS^{j},Y,l)$ is low, when $KS^{j}$ is not present in *Y*. Taking a threshold for the correlation coefficient, we can detect the presence of $\left(KS^{j}\right)$. The cross-correlation value is greater than the known signal threshold $\left(KS_{threshold}\right)$, when $KS^{j}$ is aligned to the same features in the received signal. The node makes the channel free for others in the vicinity, if the correlation value is above $KS_{threshold}$. A node goes for the transmission if it finds the cross-correlation value to be below $KS_{threshold}$, i.e., if it does not receive any signal during the CCA from other nodes present within the carrier sense range of the node.

**Figure 2:**
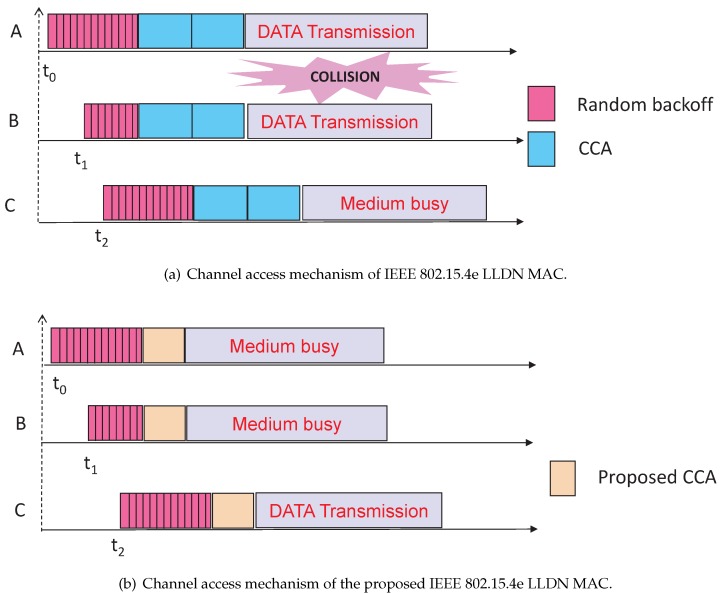
Channel access mechanism.

As shown in Figure 2a, let data packets be arrived at nodes *A*, *B* and *C* at time $t_{0}$, $t_{1}$ and $t_{2}$, respectively, where nodes *A* and *B* perform the channel access at the same slot after their random backoff. Both nodes find the channel busy due to performing CCA simultaneously. Hence, both of them should go for the random backoff. On the contrary, in IEEE 802.15.4e LLDN MAC, both nodes find the channel idle and transmit there data, which leads to a collision. Again, as shown in Figure 2b, node *C* finds the channel idle and transmits its data successfully. However, node *C* finds the channel busy in IEEE 802.15.4e LLDN MAC. Therefore, by avoiding simultaneous transmissions, our proposed channel access mechanism saves energy for nodes *A* and *B* and enhances throughput for node *C*. The detailed procedure of our proposed carrier sensing mechanism is given in Algorithm 1.
**Algorithm 1** New carrier sensing mechanism
**Require**:
Known signal KS and received signal *Y*.**Ensure**:
Channel access success/failure.1:Locate backoff period boundary, and perform *C*CA for 128 μs;2:**if** channel is found idle **then**3:    j=1;4:    Enable transmitter and transmit one random signal $KS^{k}$ from KS;5:    Receive the signal *Y*;6:    **while** j≤m **do**7:        **if** j≠k **then**8:           Calculate $C(KS^{j},Y,l)$;9:           **if** $C\left(KS^{j},Y,l\right)\;>\;KS_{threshold}$ **then**10:               Channel access failure and stop;11:           **end if**12:        **else**13:           j = j + 1;14:        **end if**15:    **end while**16:    Channel access success;17:
**end if**



### 4.3. Proposed CSMA/CA Mechanism for Shared Slots

The nodes in the network following the IEEE 802.15.4e CSMA/CA mechanism get equal chances to access the channel. For data transmission during dedicated slots $\left(SLOT_{D}\right)$ in the LLDN superframe, the slot owner $\left(SLOT_{O}\right)$ transmits data directly, whereas in the case of shared slots $\left(SLOT_{S}\right)$, nodes compete to send data by following CSMA/CA mechanism. Let the time duration of one slot, performing CCA and data transmission be $SLOT_{duration}$, $T_{CCA}$ and $T_{L}$, respectively. The nodes during the shared slots have to adopt the CSMA/CA procedure according to the recent standard. All nodes have to perform CCA two times to avoid collisions due to the transmission of acknowledgment. However, the acknowledgment for all nodes in the LLDN superframe is aggregated through one common GACK, and the use of two CCAs is not required. Hence, we suggest to modify the current channel access mechanism in order to reduce the energy consumption and latency by restricting the number of CCAs to one.

As per the current IEEE 802.15.4e standard, the node with to-be-transmitted data initializes the variables CW, NB and BE: CW represents the contention window, which depends on the random backoff value; NB represents the number of times the node has been delayed before the current transmission and is initialized to zero for each new transmission; BE represents the backoff exponent whose value increases on each channel access failure. Before transmission, the node chooses a random number in the range of $\left[0,2^{BE-1}\right]$ as backoff time periods. When this backoff period reaches zero, the node in the shared slot performs CCAs twice and starts transmission if the channel is found idle during these CCAs.

Due to the smaller slot size of the LLDN superframe, we propose here only one time random backoff before the channel access. As described in Algorithm 2, consider a node that tries to transmit data in a shared slot of the LLDN superframe. If the slot owner of the shared slot has no data to transmit, associated nodes can assess the slot through the CSMA/CA procedure. In our proposed mechanism, associated nodes have to delay for the duration of the random backoff period $\left(R_{backoff}\right)$ in the range of $\left[0,W_{S}-1\right]$ units, where $W_{S}$ is the size of the contention window for the shared slots. Let $N_{S}$ be the number of nodes having data arrival rate λ associated with the shared slot. Hence, the expected number of active nodes could be $\lambda N_{S}$. To avoid choosing the same random backoff value by the nodes, we consider $W_{S}=\lambda N_{S}$. After the random backoff period is over, the tagged node accesses the channel in the case that the transmission is possible with the current remaining time of the shared slot. Otherwise, nodes have to store their remaining backoff time at the end of the shared slot and resume the same procedure in the next shared slot.

If a channel is found idle during the CCA, a node can transmit data and waits for the ACK. The received corresponding ACK is considered as a successful transmission. However, if the node fails to receive the ACK due to collision or channel error and a retransmission slot (SLOTR) is available, it can retransmit the data. Otherwise, the packet will be discarded due to the retry failure. The retransmission slots are not assigned exclusively by the coordinator. The total numbers of retransmission slots available in the current superframe are broadcast in the beacon and the number of failed nodes through GACK by the coordinator. The node that fails first during the transmission can use the first retransmission slot. Our proposed model can reduce the number of CCAs to minimize the amount of control packet overhead.
**Algorithm 2** New channel access mechanism
**Require**:Number of sensor $N_{S}$ attached to the shared slot.**Ensure**:Transmission success/failure.1:**if** $SLOT_{S}$ and not $SLOT_{O}$ **then**2:    locate backoff period boundary, and wait for $R_{backoff}$ periods;3:    **if** ($SLOT_{duration}-R_{backoff})\;\leq \;\left(T_{CCA}+T_{L}\right)$ **then**4:        resume backoff at the next $SLOT_{S}$;5:    **else**6:        perform channel sensing as described in Algorithm 1;7:        **if** channel access success **then**8:           go to Step 14;9:        **else**10:           wait for next $SLOT_{S}$;11:        **end if**12:    **end if**13:
**else**
14:    start transmission15:    **if** transmission success **then**16:        stop;17:    **else**18:        **if** $\left(SLOT_{R}\right)$ available **then**19:           start retransmission at the allotted $\left(SLOT_{R}\right)$;20:        **else**21:           wait for next $SLOT_{S}$;22:        **end if**23:    **end if**24:
**end if**



### 4.4. Prediction Model for the Optimal Number of LLDN Retransmission Slots

The LLDN superframe of the current IEEE 802.15.4e provides retransmission opportunities to the failed nodes. However, to use these retransmission opportunities, the number of failed nodes should be less than or equal to the number of available retransmission slots in the superframe. The random allocation of the slots for retransmission by the PAN coordinator depends on the number of nodes attached to the coordinator. Due to this random allocation, it is possible that the allocated retransmission slots may be less than the number of failed nodes. If so, there is no provision in the current standard for retransmissions, and those nodes that cannot get retransmission slots in the same superframe have to reject data transmissions. Once the superframe has started, the number of retransmission slots cannot be changed. It is necessary to choose prior the number of optimal retransmission slots as correctly as possible so that we can increase the reliability. In this section, based on the number of past failed data transmissions and the allocated number of retransmission slots, we would like to find the optimal number of retransmission slots by using regression analysis. It is noted that sensors/nodes are limited in processing capacity and memory and for which we want to develop one statistical model that is used to forecast the optimal values of retransmission slots.

Let $\Psi_{1,j}$ and $\Psi_{2,j}$ be the average SINR and average waiting packets during the *j*-th LLDN superframe, respectively. The SINR value of the current superframe can be estimated by taking the mean of the lowest and highest value of the SINR that was received in the previous superframe. Let $X_{j}$ be the number of required retransmission slots during the *j*-th LLDN superframe. Considering the value $\{\left(X_{j},\;\Psi_{1,j},\;\Psi_{2,j}|j=1,2,\ldots ,n\right\}$ from the past *n* number of LLDN superframes for the prediction. We can design the regression model as:

(3) $$\hat{X_{j}}=\sum_{i=0}^{2}\vartheta_{i}\Psi_{i,j}+\delta$$

where $\hat{X_{j}}$ represents the predicted value of *X* for the *j*-th observation, $\Psi_{0,j}=1$ for j=1,2,…,n and the error term δ is added to reduce the number of packet rejections. It is to be noted that we want to minimize the error, which is the difference between the original and predicted value. In other words, we need to minimize the sum of the squared differences, i.e., $\sum_{j=1}^{n}{\left(X_{j}-\hat{X_{j}}\right)}^{2}$, where $\hat{X_{j}}$ is the predicted value of *X* for the *j*-th observation. Thus, $\sum_{j=1}^{n}{\left(X_{j}-\sum_{i=0}^{2}\vartheta_{i}\Psi_{i,j}-\delta \right)}^{2}$ needs to be minimized with three unknowns $\vartheta_{i}$ for i=0,1,2. Again, using the partial derivatives with respect to $\vartheta_{i}$ and equating to zero for each *i*, the following equation is obtained.

(4) $$\frac{\partial }{\partial \vartheta_{k}}\left(\sum_{j=1}^{n}{\left(X_{j}-\sum_{i=0}^{2}\vartheta_{i}\Psi_{i,j}-\delta \right)}^{2}\right)=0,\;\;for\;k=0,1,2.$$

Solving Equation (4), all three can be obtained. It is to be noted that the network coordinator can get the estimated number of retransmission slots required in the next superframe by using the value of $\vartheta_{i}$ in (3). Then, the coordinator modifies the superframe structure with the newly estimated retransmission slots and broadcasts the information through beacon in the next superframe. This proposed estimation can help the coordinator to allocate the optimal number of retransmission slots, which ultimately reduces the retransmission delay and increases the network reliability.

## 5. Analytical Model for Shared Slots in the LLDN Superframe

In this section, we design one Markov model to analyze the network performance on the shared slots. We consider that the network traffic is unsaturated. Based on such unsaturated traffic and the notations given in Table 1, the analytical model can be designed as follows.

**Figure 3:**
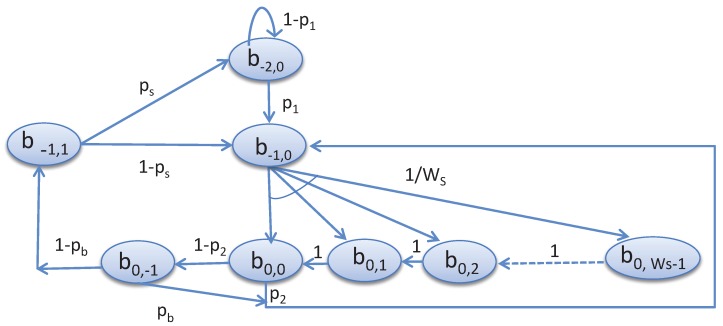
Markov models for the proposed CSMA-CA mechanism.

Recall that $N_{S}$ is the number of nodes associated with a shared slot, and each node has data arrival rate λ. As shown in Figure 3, the stochastic processes s(t) and c(t) represent the backoff states for a given node, which is fixed to zero, and the backoff counter for the contention window for the shared slot, i.e., $W_{S}$, respectively. Let, $b_{0,i}=\lim_{t\to \infty }P\left\{s\left(t\right)=0,c\left(t\right)=i\right\}$, where $i\in \{0,1,2,\ldots ,W_{S}-1\}$. The time *t* corresponds to the system time. $b_{0,-1}$, $b_{-1,1}$ and $b_{-2,0}$ represent the states corresponding to the start of the channel access, transmission and idle state, respectively. Let us consider $p_{1}$ as the probability that a node generates one packet for transmission. The node first goes to the $b_{-1,0}$ state, chooses a random backoff $b_{0,i}$ state, for $i\in \{0,1,2,\ldots ,W_{S}-1\}$. Let, $p_{b}$ be the probability of accessing the channel busy during the CCA and $p_{2}$ be the probability that the remaining time in the shared slot is sufficient to complete the data transmission. Once the channel is found idle, the node goes to the transmission state. We have considered $p_{s}$ as the probability of successful transmission. Based on the proposed Markov chain model given in Figure 3, the transition probabilities for deducing the steady state probabilities can be derived as follows.

(5) $$b_{0,i}|b_{0,i+1}=1\;\;for\;0\leq i\leq W_{S}-1.$$

(6) $$b_{-1,0}|b_{0,-1}=p_{b}.$$

Equation (5) represents the decrement of the backoff counter, which occurs with a probability of one. Equation (6) represents the probability of finding the channel busy during the CCA, and thereafter, a node selects a state in the next backoff state. Based on these transition probabilities, we can derive the steady state probabilities as follows. A node normally goes for the CCA after the random backoff period whenever the remaining time in the shared slot is enough to transmit the data. The corresponding steady state probability can be deduced as follows.

(7) $$b_{0,-1}=\left(1-p_{2}\right)b_{0,0}.$$

If the remaining time in the shared slot is sufficient for the data transmission, the node goes for the CCA, and upon finding the channel idle, it starts the data transmission. The corresponding state probability is:

(8) $$b_{-1,1}=\left(1-p_{b}\right)b_{0,-1}.$$

When a transmission failure occurs without exceeding the retransmission limits, the tagged node enters into the active state. The corresponding steady state probability can be deduced as follows.

(9) $$b_{-1,0}=\left(1-p_{s}\right)b_{-1,1}+p_{1}b_{-2,0}+\left(p_{2}+\left(1-p_{2}\right)p_{b}\right)b_{0,0}.$$

The node enters into the idle state after successful data transmission or no packet to transmit. The corresponding steady state probability is:

(10) $$b_{-2,0}=\frac{1}{p_{1}}\left(p_{s}b_{-1,1}\right).$$

By the theory of total probability of the Markov model, we have the equation:

(11) $$b_{-2,0}+\sum_{i=-1}^{W_{S}-1}b_{0,i}+\sum_{k=0}^{1}b_{-1,k}=1$$

Let ϕ be the probability that a node attempts to do carrier sensing. Then, this probability can be as follows.

(12) $$\phi =b_{0,-1}.$$

Normally, there are two cases if the channel is found busy by the node. The channel may be busy when another node present within its sensing range is transmitting or any other node also accessing the channel in the same slot and is transmitting the known signal. During one beacon interval, the probability of a node having data arrival is $1-e^{-\lambda BI}$. Therefore, the expected number of nodes $\left(N_{E}\right)$ with data arrival associated with a shared slot will be:

(13) $$N_{E}=N_{S}\left(1-e^{-\lambda BI}\right).$$

Hence, the value of the probability of the channel being busy (α) due to simultaneous CCAs and the probability of the channel being busy (β) due to data transmission by any other node can be calculated as follows.

(14) $$\alpha =1-{\left(1-\phi \right)}^{N_{E}-1}.$$

(15) $$\beta =\frac{N_{E}\phi {\left(1-\phi \right)}^{N_{E}-1}}{1-{\left(1-\phi \right)}^{N_{E}}}.$$

Hence, the probability of a busy channel during CCA for the node is:

(16) $$p_{b}=\alpha +\left(1-\alpha \right)\beta .$$

## 6. Performance Analysis

The bit error rate $P_{b}\left(\varsigma \right)$ for the IEEE 802.15.4 radios is defined in [1] as:

(17) Pb(ς)=0.033∑i=216(−1)i16ie20ς(1i−1)

where ς is the instantaneous SINR. A node after generating the packet will access the channel before entering the transmission state. The transmission in the shared slots will be successful if only one node accesses the channel successfully without any interference or link error. Hence, the probability of the successful transmission in the shared slot is:

(18) $$P_{TSSS}=\frac{N_{E}\phi {\left(1-\phi \right)}^{N_{E}-1}}{1-{\left(1-\phi \right)}^{N_{E}}}{\left(1-P_{b}\left(\varsigma \right)\right)}^{L_{data}}$$

where $L_{data}$ is the length of the data packet in bits and $N_{E}$ is the expected number of nodes attached to the concerned shared slot.

### 6.1. Reliability

Let the LLDN superframe contain $M_{1}$ number of transmission slots and $M_{2}$ number retransmission slots. According to IEEE 802.15.4e, if one transmission has failed in the *k*-th slot, the tagged node retransmits the data in the same superframe provided a retransmission slot is available. A failed node gets a retransmission slot, if either $\left(k-1\right)<M_{2}$ or the total failed nodes before the *k*-th slot is less than $M_{2}$. Therefore, the probability that the node transmitting in the *k*-th slot may get a retransmission slot is:

(19) $$P_{RS}=\left\{\begin{array}{c} 1,\;if\;\;Max\{k-1,number\;of\;failure\\ \;nodes\;before\;k-th\;slot\}<M_{2}\\ 0,\;otherwise. \end{array}\right.$$

In the case of any transmission failure, the tagged node retransmits the data in the allotted retransmission slot after getting the transmission failure information in GACK with the available retransmission slot. However, in this slot, only the collided nodes will access the channel. Let the expected number of collided nodes be $N_{c}$. Therefore, the retransmission successful probability of the node is given as follows.

(20) $$P_{RTS}=\frac{N_{c}\phi {\left(1-\phi \right)}^{N_{c}-1}}{1-{\left(1-\phi \right)}^{N_{c}}}{\left(1-P_{b}\left(\varsigma \right)\right)}^{L_{data}}.$$

In the LLDN superframe, a transmitted packet by a node to the coordinator is consider as successful when the transmission or retransmission in the allocated slot is successful. Therefore, the successful transmission probability for a node in the LLDN superframe is given as follows.

(21) $$P_{success}=P_{TSSS}+\left(1-P_{TSSS}\right)P_{RS}P_{RTS}.$$

### 6.2. Throughput

Network throughput is defined as the average successful data rate over the communications link. The maximum throughput can be achieved in the network when exactly one node transmits the data to the destination. The data communication during the shared slot in the superframe is through the slotted CSMA/CA mechanism. The throughput of the IEEE 802.15.4e network can be calculated as:

(22) $$Throughput=L_{pkt}P_{success}PHY_{rate}$$

where $L_{pkt}$, $P_{success}$, $PHY_{rate}$ are the payload duration, the probability of successful packet transmission and the physical data rate of IEEE 802.15.4e, respectively.

### 6.3. Energy Consumption

In this section, we analyze the average energy consumption of one node to transmit data successfully in shared slots of the superframe. Taking $T_{L}$ as the time required for transmitting a packet and $\left(T_{CCA}\right)$ for each successful channel assessment, the average energy consumption for a node can be calculated as:

(23) $$\begin{array}{c} Average\;Energy=\alpha T_{CCA}P_{rx}+\left(1-\alpha \right)T_{S}P_{tx}\\ \;\;\;\;\;\;\;\;\;+\left(1-p_{b}\right)T_{L}P_{tx} \end{array}$$

where $P_{rx}$, $P_{tx}$ are the energy consumption to receive and transmit a packet, respectively. $T_{S}$ is the time duration of transmitting the known signal.

### 6.4. Delay Analysis

The network delay is defined as total time taken for the generated information to travel when a communication between two nodes in a network takes place. In this paper, we consider the waiting time ($W_{time}$), propagation delay ($P_{delay}$) and transmission delay ($T_{delay}$).

- $W_{time}$ is the waiting time for a device until the next shared slot if the packet is generated outside the allotted shared slot period.
- $P_{delay}$ in a network is the amount of time needed for one bit of data to travel to a one hop destination.
- $T_{delay}$ is defined as the amount of time required to transmit the total payload with respect to the link data rate.

Therefore, the average transmission delay in the IEEE 802.15.4e network, when transmission is successful in the *j*-th superframe, can be calculated as:

(24) $$\begin{array}{c} Delay=\sum_{j=1}^{\infty }{\left(1-P_{success}\right)}^{j-1}P_{success}(\left(j-1\right)BI\\ \;\;\;\;+W_{time})\;+T_{delay}+P_{delay} \end{array}$$

where $P_{success}$ is the successful data transmission probability and BI is the beacon interval.

## 7. Simulation Results

In this section, we describe our simulation results and validate the models using the OMNeT++ [34] simulator. We conduct the simulation to compare the performance of our protocol with IEEE 802.15.4e. We have analyzed the performance of our proposed protocol under the assumption that nodes are active in their allotted slots and go for power saving mode in the rest of the beacon interval. In our simulation environment, the star topology is considered, and the nodes are deployed randomly, keeping the coordinator in the center of the circle. We set the simulation parameters according to the IEEE 802.15.4e standard, which are given in Table 2.

**Figure 4:**
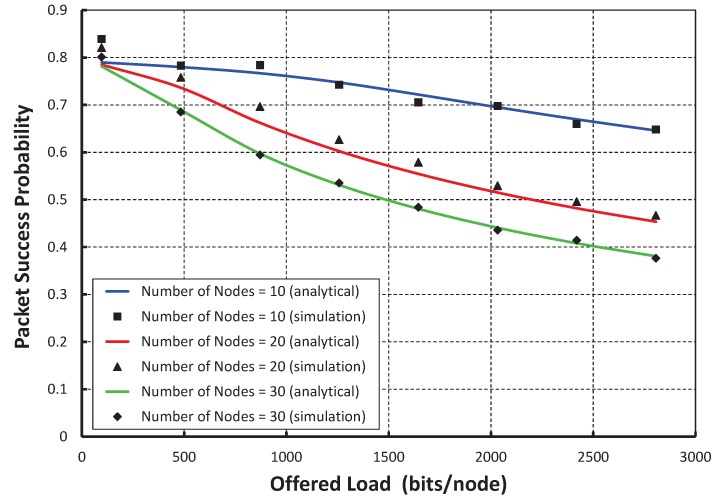
Validation for packet success probability with different offered load.

**Figure 5:**
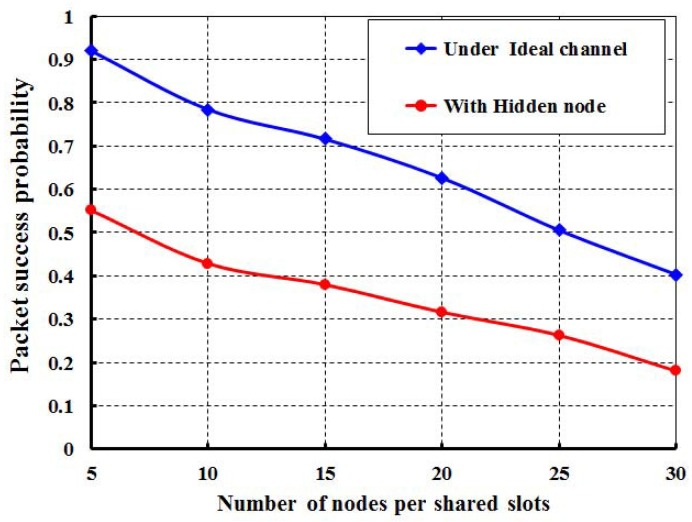
packet success probability with and without hidden nodes.

**Figure 6:**
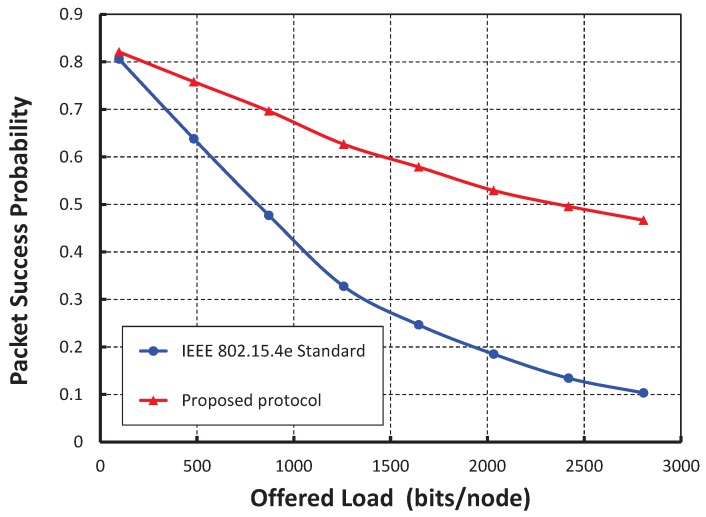
Comparison of our protocol with IEEE 802.15.4e.

In Figure 4, let the *X* axis be the different offered loads for each node and the *Y* axis be the corresponding packet success probabilities. It is observed that the packet success probabilities decrease with respect to the loads per node increase irrespective of the number of nodes in the network attached to a shared slot. When 10 nodes per shared slot are considered and the offered load is of 500 bits, we find that the success probability is 0.77. However, we got the success probabilities for 20 and 30 nodes per shared slots as 0.71 and 0.68, respectively. It is also seen from Figure 4 that the simulation result matches with the analytical one. From Figure 5, it is observed that the packet success probabilities decrease with respect to the number of nodes attached to the shared slot with or without hidden terminal problem under the offered load 200 bits/node. The comparison of our proposed protocol with IEEE 802.15.4e for the packet success probability under ideal channel condition with 20 nodes per shared slots is shown in Figure 6. It is observed that the packet success probabilities decrease, if the offered loads to a shared slot increase. According to the IEEE 802.15.4e standard, the initial contention window for the stations is [0,8]. Hence, the expected number of nodes choosing the same slot will be 20×(1/8)≥2. Accordingly, the chance of transmission by two stations at the same time is higher. Fortunately, in our proposed protocol, the contention window is based on the active number of nodes per shared slot such that the expected number of nodes choosing the same slot is always less than or equal to one. Hence, the packet success probability of our MAC protocol outperforms, as noticed in Figure 6. Even if, two nodes choose the same random backoff value and access the channel in the same slot, our carrier sensing mechanism lets the nodes go to the backoff state. For example, as shown in Figure 2b, *A* and *B* find the channel busy due to simultaneous CCAs and stop the packet transmissions, and *C* can transmit its packet successfully. As a result, the packet success probability is enhanced.

**Figure 7:**
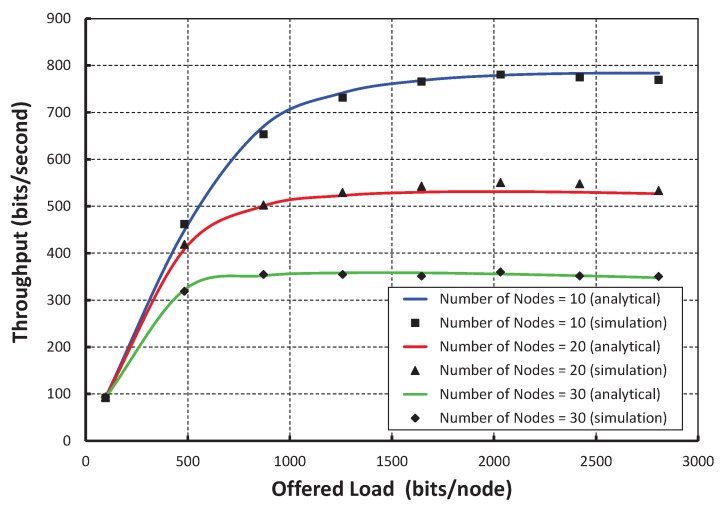
Validation of throughput for different offered loads.

**Figure 8:**
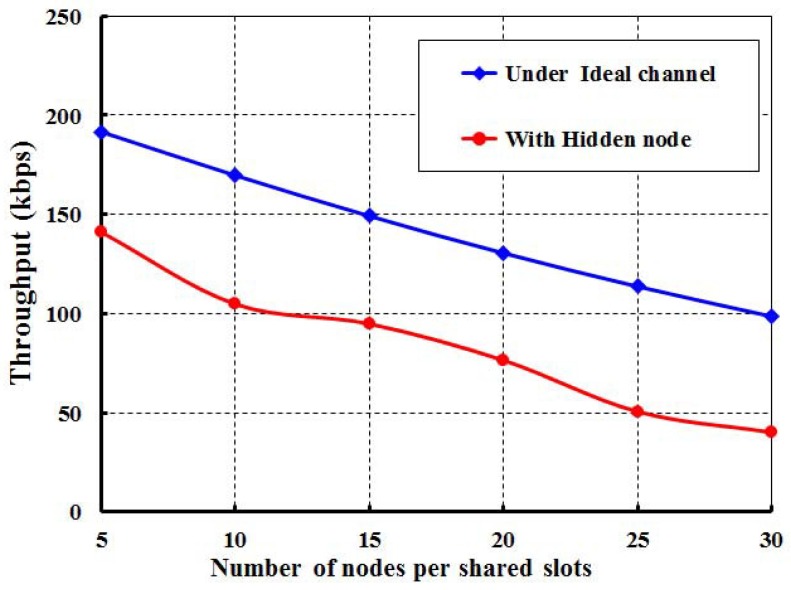
Throughput with and without hidden nodes.

**Figure 9:**
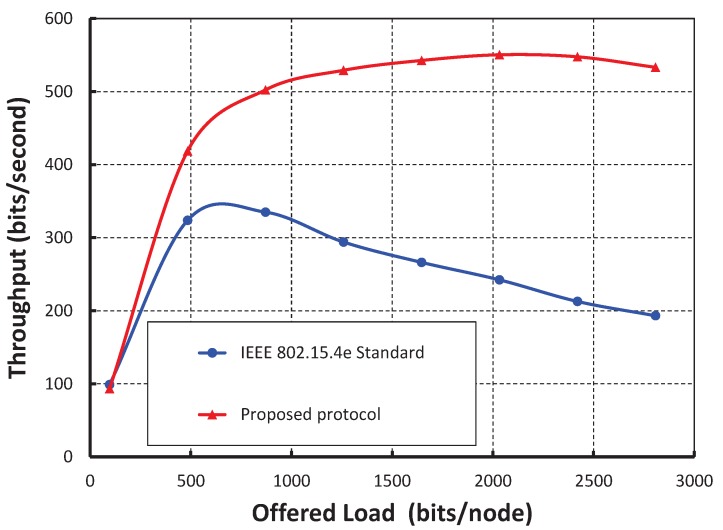
Comparison of throughput of our protocol with IEEE 802.15.4e.

In Figure 7, we consider the offered loads along the *X* axis and the corresponding throughput along the *Y* axis. The data payload size is a maximum of 127 bytes including the physical and MAC layer overhead and address. We consider the physical, MAC layer and address to be 6 bytes, 3 bytes, and 8 bits, respectively. The throughput initially increases with respect to the offered load. However, it remains constant after certain offered loads. The significant changes in the graph are due to the presence of different numbers of nodes per shared slot. We observe that the maximum throughput is achieved as 780 bits per second, when only 10 nodes per shared slots are considered and the payload is 100 bytes. However, the throughput remains constant after the offered load is 800 bytes irrespective of the number of nodes. The close agreement between the simulation and analytical results shows the accuracy of the proposed analytical model though there is little difference due to the assumptions. From Figure 8, it is shown that throughput decreases with respect to the number of nodes attached to the shared slot with or without the hidden terminal problem considering the offered load as 200 bits/node. As shown in Figure 9, we compare the IEEE 802.15.4e protocol with ours. It is observed that the throughput of our protocol is significantly higher than the standard when we consider only 20 nodes per shared slot. This happens since our carrier sensing mechanism sends the nodes to backoff when they access the channel in the same time slot. Hence, any other node can access the channel successfully.

**Figure 10:**
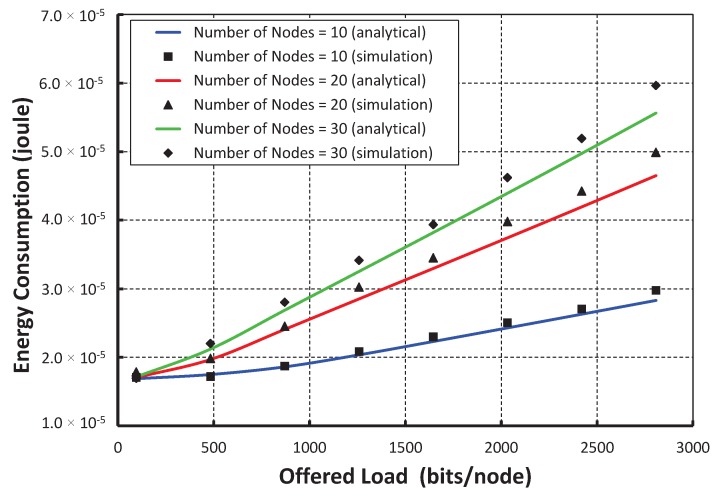
Validation of energy consumption for different offered loads.

**Figure 11:**
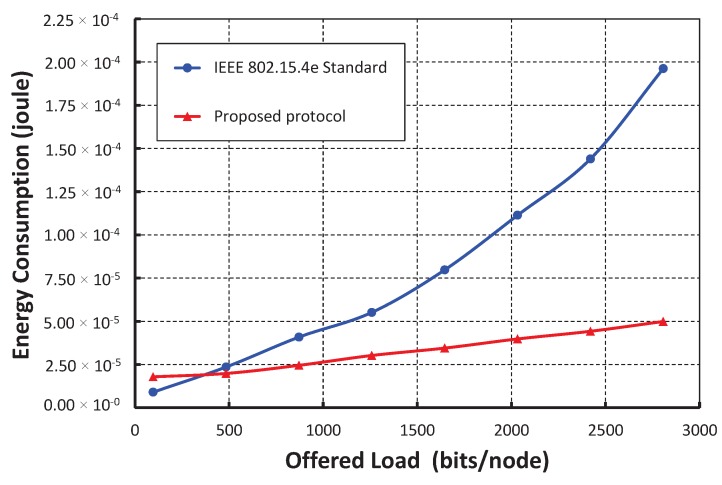
Comparison of the energy consumption of our protocol with the IEEE 802.15.4e standard.

As shown in Figure 10, we take the offered loads along the *X* axis and get the average energy consumption along the *Y* axis. The energy consumptions for data transmissions in a shared slot increase with respect to the loads per node increase, which is due to the active number of nodes presented in a shared slot. As shown in Figure 11, we compare our proposed protocol with the IEEE 802.15.4e standard. It is observed that the energy consumption in our protocol is significantly less than the standard when only 20 nodes per shared slot are considered. This result happens as our carrier sensing mechanism sends one additional known signal that lets the nodes accessing the channel in the same slot to go for backoff. For example, as shown in Figure 2b, *A* and *B* find the channel busy as they perform simultaneous CCAs and stop the packet transmissions. Hence, both nodes *A* and *B* can save energy in our protocol.

**Figure 12:**
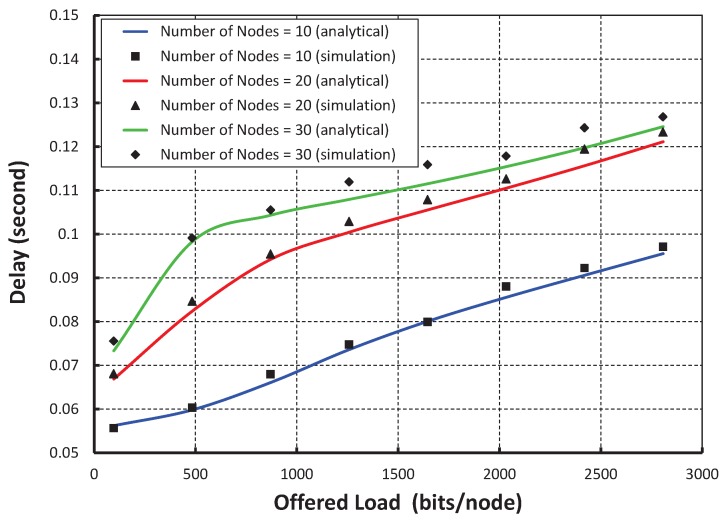
Validation of transmission delay for different offered loads.

**Figure 13:**
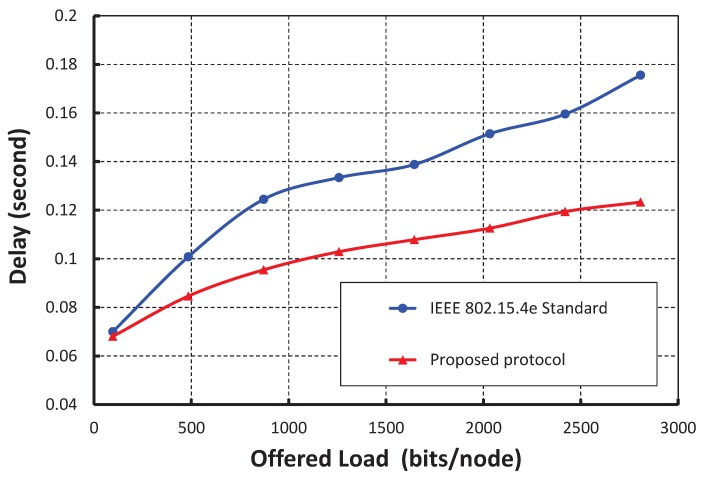
Comparison of the transmission delay of our protocol with the IEEE 802.15.4e standard.

Figure 12 shows the result of transmission delay for a data payload of 100 bytes. It is observed that the transmission delay increases with the increase in the offered loads irrespective of the number of nodes accessing in a shared slot. As shown in Figure 13, the transmission delay in our protocol is compared with the IEEE 802.15.4e standard under the ideal channel condition. It is observed that the transmission delay of our protocol is significantly less as compared to the IEEE 802.15.4e standard.

**Figure 14:**
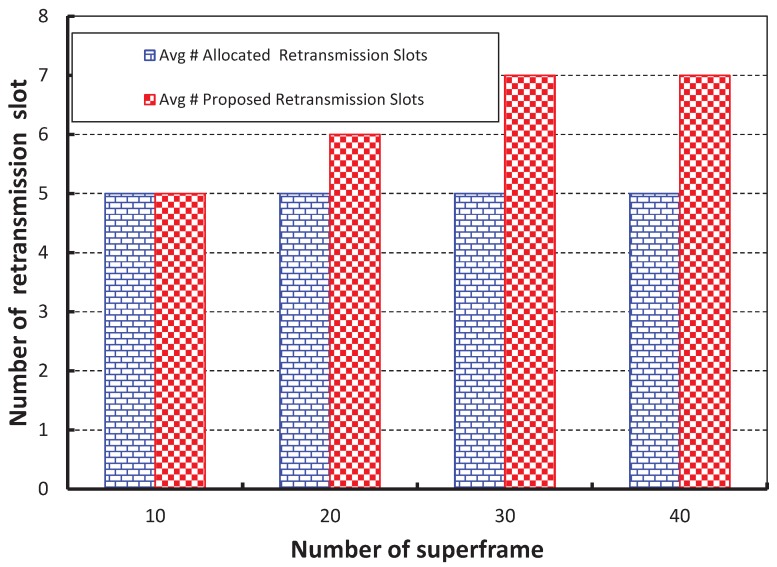
Optimal number of retransmission slots for different numbers of superframes.

In Figure 14, we take the number of superframes along the *X* axis and get the expected number of retransmission slots along the *Y* axis. It is observed that the required average number of retransmission slots is nearly equal to our predicted value when 10 superframes and 20 nodes per shared slot are considered. However, the predicted number of retransmission slots is higher than the average number of retransmission slots when more superframes are considered.

**Figure 15:**
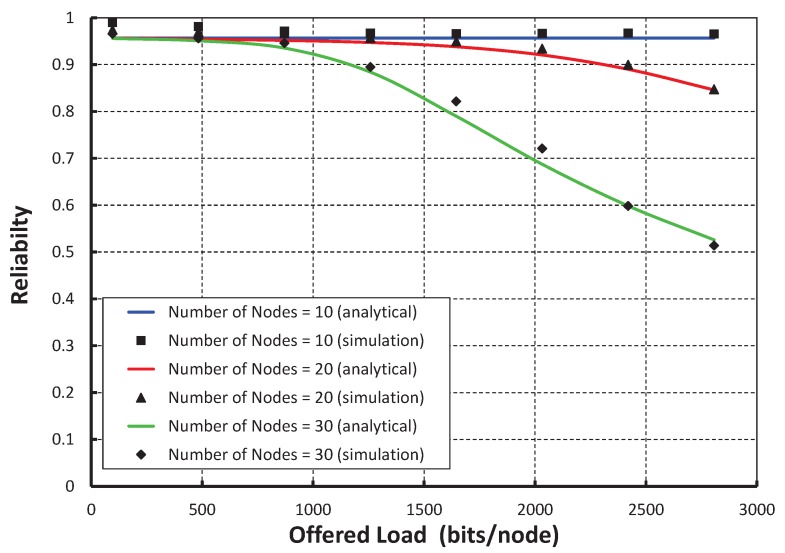
Validation of the reliability for different offered loads.

**Figure 16:**
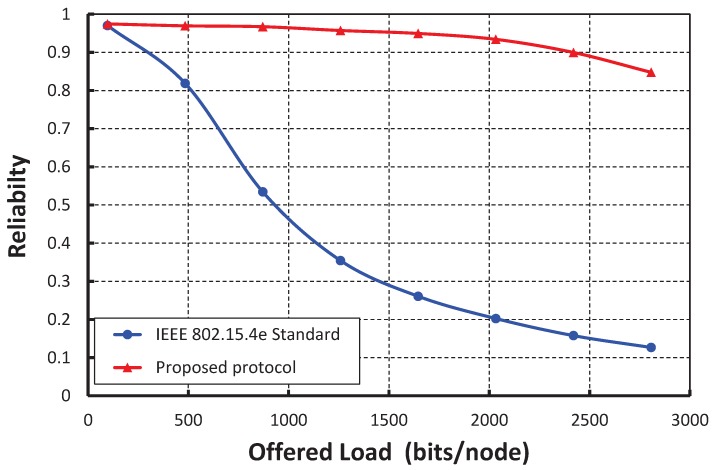
Comparison of reliability of our protocol with the IEEE 802.15.4e standard.

Figure 15 shows the result of reliability for different offered loads. We find that the radiabilities decrease with respect to the offered loads and irrespective of the number of nodes accessing in a shared slot. In Figure 16, we compare the reliability of the IEEE 802.15.4e standard with ours under the ideal channel condition. It is observed that the reliability of our protocol is significantly higher than the IEEE 802.15.4e standard. Table 3 shows the summarization of the statistical results for transmission success probability, throughput, delay, energy consumption and reliability.

## 8. Conclusions

In this paper, we propose a new CSMA/CA mechanism for the IEEE 802.5.4e LLDN superframe to reduce the number of CCAs. In order to improve the efficiency of the system, a prediction model is proposed to predict the optimal number of retransmission slots so that the possibility to retransmit a failed packet can be guaranteed. We present a novel medium access algorithm that exploits two radios such that it reduces the number of simultaneous transmissions, thus leading to lower delay and overall energy consumption. Performance analysis for data transmission reliability, throughput and energy consumption are made based on our proposed models. Our designs are validated with the simulation and analytical methods, which are well matched. Performance comparisons between the proposed solution and the original 802.15.4e LLDN MAC protocol have been done and show that our design protocol is better than the IEEE 802.15.4e MAC in terms of throughput, energy consumption, delay and reliability. Hence, our design can be implemented in WSNs for different applications like fire detection and alarm systems, fall detection systems, factory automation, robots, automated dispensations, airport logistic and many more emergent automated applications.

## Figures and Tables

**Table 1:** Notation table.

Notation	Meaning
*N*	Total number of nodes in a system.
*L*	Length of a data packet.
GACK	Group acknowledgment.
λ	Packet arrival rate.
$R_{backoff}$	Random backoff.
$p_{1}$	Probability that a node has a packet to transmit.
$p_{2}$	Probability that remaining time slots are sufficient to transmit a packet.
$p_{b}$	Probability that the channel is busy during a CCA.
ϕ	Probability that a node is sensing the channel first time.
α	Probability that the channel is busy due to simultaneous CCA.
β	Probability that the channel is busy due to data transmission.
$P_{b}\left(\varsigma \right)$	Probability of channel bit error rate.
$P_{TSSS}$	Probability of transmission success in a shared slot.
$P_{RS}$	Probability of getting a retransmission slot.
$P_{RTS}$	Probability of retransmission success.
$P_{success}$	Probability of transmission success within an LLDN superframe.
$L_{beacon}$	Length of the beacon packet in bits.
$L_{data}$	Length of the data packet in bits.
$L_{GACK}$	Length of the group acknowledgment packet in bits.
$T_{CCA}$	Duration of CCA.
$T_{L}$	Duration of data transmission.
$SS_{duration}$	Duration of one shared slot.

**Table 2:** Simulation parameters.

Notation	Meaning
Parameters	Value
Radio band	2.4 GHz
Channel bandwidth	250 kbps
Carrier sense sensitivity	−85 dBm
Channel number	11
Superframe duration	31.2 ms
Number of nodes per shared slot	20
Shared slot duration	1.95 ms
Number of transmission slots in one superframe	10
Number of retransmission slots in one superframe	5
Unit backoff period	20 symbol
PHY overhead	6 byte
MAC overhead	3 byte
Short address	8 bit
Transmission current consumption	9.1 mA
Receiving current consumption	5.9 mA
Sleep current consumption	0.001 mA

**Table 3:** Summarization of the obtained result of our protocol with the IEEE 802.15.4e standard.

Offered Load (bits/node)		500	1000	1500	2000	2500
**Packet Success**	IEEE 802.15.4e Standard	0.618656	0.390007	0.280493	0.196157	0.133287
**Probability**	Proposed protocol	0.765746	0.689023	0.617008	0.540302	0.503295
**Throughput**	IEEE 802.15.4e Standard	344.1575	331.1715	282.2049	246.1682	210.906
**(bits/s)**	Proposed protocol	475.9602	541.2176	542.543	567.9403	558.6206
**Delay (s)**	IEEE 802.15.4e Standard	0.11381	0.13283	0.138705	0.152111	0.17582
	Proposed protocol	0.089035	0.103028	0.10966	0.115764	0.124364
**Energy Consumption**	IEEE 802.15.4e Standard	3.11 × 10${}^{-5}$	4.9 × 10${}^{-5}$	7.39 × 10${}^{-5}$	11.3 × 10${}^{-5}$	16.7 × 10${}^{-5}$
**(joule)**	Proposed protocol	2.5 × 10${}^{-5}$	3.23 × 10${}^{-5}$	4.31 × 10${}^{-5}$	4.43 × 10${}^{-5}$	5.28 × 10${}^{-5}$
**Reliability**	IEEE 802.15.4e Standard	0.782367	0.415697	0.283299	0.195581	0.145791
	Proposed protocol	0.940286	0.937278	0.927357	0.922573	0.889301
